# Nursing and Midwifery's Impact on Health Policy Development: A Literature Review

**DOI:** 10.1111/inr.70061

**Published:** 2025-07-16

**Authors:** David Stewart, Kathleen Baird, Jill White, James Buchan

**Affiliations:** ^1^ Faculty of Health University of Technology Sydney, Australia International Council of Nurses Ultimo Australia; ^2^ School of Nursing and Midwifery Faculty of Health University of Technology Sydney Ultimo NSW Australia; ^3^ Faculty of Health University of Technology Sydney Ultimo NSW Australia

**Keywords:** Barriers, health policy, leadership, literature review, midwives, nurses, policy development, professional autonomy, workforce development

## Abstract

**Aim:**

This study explores how nursing and midwifery can enhance their influence on health policy development and identifies strategies to optimize their impact on global health systems.

**Background:**

Nurses and midwives, the largest group of healthcare professionals, play vital roles in healthcare delivery but remain underrepresented in health policy development. Historical barriers, gendered power dynamics, and systemic limitations hinder their meaningful involvement, despite their critical role in care provision. Addressing these issues is crucial to unlocking their potential to improve health systems worldwide.

**Methods:**

A literature review was conducted, analyzing research, policy reports, editorials, and commentaries from 2013 to 2023. Key barriers, enablers, and strategies to enhance nursing and midwifery's role in health policy were identified through database searches and reference checks, with thematic analysis used to synthesize findings.

**Findings:**

The review identified several barriers to nursing and midwifery's involvement in health policy, including power imbalances, limited policy education, and insufficient organizational support. Current literature often focuses on individual experiences but overlooks broader systemic factors, such as the roles of key policy actors, regulations, and technologies. Strategies to enhance participation include integrating health policy into nursing curricula, leadership programs, and strengthening professional associations. A more nuanced, multifaceted approach is needed to address these barriers and encourage policy engagement.

**Discussion:**

To empower nurses and midwives, future research should adopt frameworks like Actor Network Theory to consider the broader context of health policy development. Strategies must focus on education, tackle power imbalances, address gender dynamics, and promote collective action through professional organizations.

**Conclusion and Implications for Nursing and/or Health Policy:**

Nurses and midwives must be recognized as essential contributors to health policy. By addressing systemic barriers and implementing comprehensive strategies, nursing and midwifery can shape policies that reflect care delivery realities and improve global health outcomes. Future research should adopt a broader approach, incorporating the full spectrum of actors and systemic factors in health policy development.

## 1 Introduction

The World Health Organization (WHO) (2024) estimates that there are 29 million nurses and 2.2 million midwives worldwide, constituting approximately 60% of the health professions and the largest occupational group within the health sector. Optimizing the use of this workforce can transform health systems so that patients are able to access quality and affordable care. Throughout the world, nurses and midwives are leading and contributing to innovations to improve health outcomes (World Health Organization 2024). However, there are often a variety of historical, regulatory, funding models, and policy barriers that limit nursing and midwifery's ability to maximize their contribution that would enable high‐performing health systems (Stewart 2022). An optimized and empowered nursing and midwifery workforce can facilitate a more accessible, affordable, safer, and sustainable healthcare system (National Academies of Sciences 2021).

High‐quality care demands constant adaption to an evolving consumer, community, and government needs. Countries face long‐term challenges (e.g., aging societies and increasing chronic conditions), increasing demands for services, critical workforce shortages, growing inequalities, and tightening public finances (Stewart et al. 2019). Complex decisions must advance health system performance, improve patient outcomes, reduce inequalities, and achieve greater value for money. This requires a culture of innovation and rapid implementation of good practices and ideas. Therefore, nurses and midwives should play a central role in leadership (National Academies of Sciences 2021).

Given their potential to transform health systems, examining their contribution to policy development is crucial. This literature review examines the various ways in which nursing and midwifery can enhance their influence on shaping quality and effective health policy to improve health systems. The literature review examines existing research and evidence, exploring the challenges, opportunities, and strategies for nursing to become more involved in policy development.

## 2 Background

WHO (2018) describes health policy as the decisions, plans, and actions that are undertaken to achieve specific healthcare goals within a society. This entails the creation of frameworks that will govern the planning and delivery of health care services, as well as the determination of priorities, the allocation of resources, and the monitoring of performance. The term “health policy” refers to a broad concept that includes a variety of subtopics, such as financing, the provision of services, the management of human resources, and the governance of health systems. They exist at all levels, including societal, organizational, regional, and local (Anderson et al. 2022).

Health policy shapes a country's healthcare system and influences the quality, affordability, and accessibility of health services, affecting population health outcomes (World Health Organization 2018). Policies can effectively address health disparities and protect vulnerable populations. As a result, policies can have immediate to long‐lasting effects on the health and well‐being of individuals, communities, and populations.

WHO (2018) has provided guidance on the development of health policies that improve the quality of care. They recognize that evidence‐informed policy development is crucial to ensure that health policies are grounded in scientific evidence and are responsive to the needs and priorities of the population. In addition to this, they emphasize the important role of engaging with key stakeholders to develop policy that underpins good‐quality health services.

Walt and Gilson (1994) articulated that effective health policy is often not implemented and evaluated effectively because there is a focus on research, neglecting actors, context, and processes. They also recognize that the development of health policy is a complex and a dynamic process requiring collaboration among various stakeholders, including government, healthcare providers, civil societies, communities and health professionals. They developed a policy analysis framework specifically for health policy, addressing the need for a more comprehensive approach to understanding the development and implementation of health policies. This framework, known as the policy triangle, emphasizes the critical role of actors, context, and processes in addition to the content of the policy. By neglecting key actors, many health policies will fail, as these entities are often responsible for developing and/or implementing the policies. Understanding the impact of nursing and midwifery actors in the policy process can provide valuable insights for developing and implementing effective health policies. This literature review seeks to explore the research related to nursing and midwifery actors and their impact on health policy.

## 3 Methods

Literature, including academic journal articles and gray literature, was identified using PubMed. The search was conducted on November 3, 2023. Table 1 outlines the search terms and strategy for the search.

**TABLE 1:** Search terms and strategy for the search.

Search topic	How can the nursing profession improve its influence on shaping quality and effective health policy?
Key terms	Nursing; national nursing associations; health policy, policies, participation, barriers, benefits
Date range	Last 10 years
Study type	Any study type
Age range	All ages
PubMed, includes MeSH	(“Nurses”[Mesh] OR “Nursing”[Mesh] OR “Nurse's Role”[Mesh] OR “Midwifery”[Mesh] OR “Nurse Midwives”[Mesh] OR “nurse”[ti] OR “nurses”[ti] OR “nursing”[ti] OR “midwife”[ti] OR “midwives”’[ti] OR “midwifery”[ti] OR “nurse midwife”[ti] OR “nurse midwives”[ti] OR “nurse‐midwife”[ti] OR “nurse‐midwives”[ti]) AND (“Health Policy”[Mesh] OR ((“health”[tiab] OR “healthcare”[tiab]) AND (“Policy”[Mesh] OR “Public Policy”[Mesh] OR “policy”[ti] OR “policies”[ti]))) AND (“influence”[tiab] OR “influencing”[tiab] OR “advocacy”[tiab] OR “participation”[tiab] OR “involvement”[tiab] OR “role”[tiab] OR “leadership”[tiab] OR “strategic”[tiab] OR “strategy”[tiab] OR “voice”[tiab] OR “engaging”[tiab] OR “engagement”[tiab] OR “engage”[tiab] OR “representation”[tiab] OR “representative”[tiab] OR “representatives”[tiab] OR “improve”[tiab] OR “improvement”[tiab] OR “improvements”[tiab] OR “benefits”[tiab] OR “barriers”[tiab] OR “effective”[tiab] OR “effectiveness”[tiab]) AND 2013:2023[dp]

### 3.1 Screening Process

**FIGURE 1:**
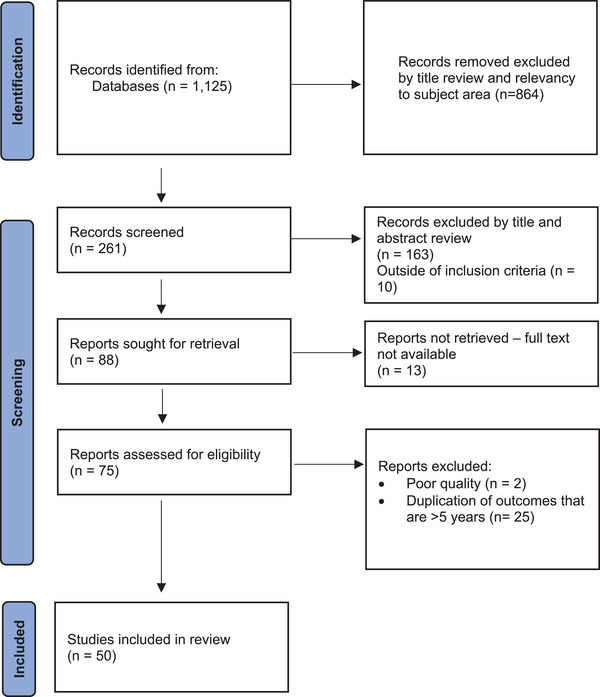
PRISMA flow diagram.^1^

For this review, articles were selected based on specific inclusion and exclusion criteria, focusing on studies published in the last 10 years (2013–2023) and encompassing qualitative research and gray literature. The aim was to prioritize studies that delve deeply into the experiences, barriers, and strategies related to nursing and midwifery's influence on health policy. A summary of the screening process is shown in Figure 1.

### 3.2 Selection Criteria

1. Inclusion criteria:
  - Studies involving nurses' and midwives' roles, contributions, and impact on health policy.
  - Qualitative studies and systematic literature reviews that explore in‐depth factors such as barriers and facilitators for nursing and midwifery in health policy advocacy.
  - Gray literature sources such as policy reports, case law, legislation, and government publications to provide additional insights.
  - Articles focused on nurses and midwives from various regions and healthcare settings to capture a global perspective.
  - Research published in English within the last decade (2013–2023) to ensure contemporary relevance and applicability.
2. Exclusion criteria:
  - Non‐English articles, conference abstracts, or studies lacking in qualitative depth.
  - Articles not focused on nursing and midwifery's roles in health policy (e.g., those that primarily address clinical care without a policy component).
  - Due to limitations with referencing, duplicates of findings >5 years were removed.

### 3.3 Screening and Quality Consideration

All articles were initially imported into a reference management system (Endnote), where duplicates were removed. The remaining documents were screened for alignment with the inclusion criteria and their relevance to the research topic. The screening process was conducted by DS, who reviewed each article for its adherence to the predefined inclusion and exclusion criteria. Literature quality was assessed based on the clarity of writing and the relevance of the reported outcomes. Quality assurance was conducted by the other reviewers. In cases of disagreement about the inclusion of certain literature, conflicts were resolved through discussions among all reviewers, with one of the reviewers (KB) nominated to be the decision‐maker if consensus could not be reached. However, this was not necessary as a consensus was always reached.

To comply with journal restrictions, documents older than five years with duplicated outcomes were excluded. The final selection of documents was categorized by primary focus areas, research methodologies, and themes relevant to nursing and midwifery's engagement in health policy. This process ensured that only the most relevant literature was included in the review.

## 4 Findings

### 4.1 Nursing and Midwifery's Impact on Health Policy

The literature underscores the importance of nursing and midwifery's contribution to health policy development. The most common reason cited is that nurses and midwives form the largest group of healthcare professionals (Hajizadeh et al. 2021; Benton et al. 2020). While nurses and midwives form the largest group of healthcare professionals, their sheer numbers alone do not justify their involvement in policy development. ICN (Stewart et al. 2021) provides clearer guidance on the importance of the involvement of nursing in the development of health policies. They state that nurses have a unique position among health care providers. They have a critical role in the provision of health care across the continuum and spend more time than any other profession with consumers requiring healthcare. This is supported by Salvage and White (2019), who emphasize that nurses, as the largest group in the global health workforce, occupy a “special position as the interface between the health system and the community, and they see, hear and know how policy and politics affects patients and communities” (. 148). As a result of this position, nurses have invaluable knowledge and insight that can improve the quality and efficiency of health policy. Aribi et al (2014) similarly argue that nurses’ influence in health polices improves patient safety, increases quality of care, and elevates occupational safety.

Disch (2020) points out that policies developed without nursing input are often ‘impractical, expensive, unrealistic and not followed.’ This was part of the finding of the United States of America Senate's Subcommittee on Primary Health and Aging (Than 2014) from a written testimony which stated:

The question that nurses often ask is “Does this work at 2:00 a.m.?” We have a very pragmatic appreciation for what works round the clock and on weekends. We often have solutions for seemingly intractable system issues or personal situations. (. 2)

The value that nursing and midwifery brings to the table is not just because of the sheer numbers but also because of the way that they view the world, which Disch (2020) calls the “nursing lens.” They state that:

…the nursing lens offers more than a way to characterize the contributions of nurses. Because of the way that nurses think, view situations holistically, engage diverse stakeholders, craft pragmatic yet innovative solutions, and understand the human condition… this perspective is vitally needed in boardrooms, at policy tables and in senior leadership positions. (. 17)

Extensive research supports the role of nursing and midwifery in shaping high‐quality health policies. Organizations such as ICN, ICM, and WHO argue for their involvement based on their unique “nursing and midwifery lens.” With direct interactions and practical insights into care delivery, nurses and midwives are well‐positioned to bridge gaps between needs, policy creation, and implementation, offering solutions from bedside care to systemic issues (Salvage and White 2020). Recognizing this perspective leads healthcare organizations to increasingly advocate for integrating nurses and midwives into leadership roles, policy discussions, and governance boards (Salvage et al. 2019b). As Mason et al. (2013) point out, such inclusion should not merely be a gesture, toward the profession, but rather an essential stride toward attaining appropriate, quality, and efficient healthcare policies that are firmly rooted, in the practicality of community health needs and the delivery of healthcare services.

### 4.2 The Barriers to Nurses and Midwives’ Participation in Health Policy Development

The lack of influence of nurses and midwives in health policy development is consistent across various socioeconomic, demographic, and cultural contexts, as indicated in research from Iran, Kenya, Jamaica, South Africa, Uganda, Tanzania, the USA, the UK, and Australia (Hajizadeh et al. 2022; Etowa et al. 2023; Haney 2022; Hannigan 2022). Although there are many differences across the world in terms of the health policymaking process, there are consistent themes. These relate to societal factors, the work environment and organizational factors, and nursing and midwifery‐related factors (Table 2).

**TABLE 2:** Key themes and subthemes of barriers to nurses and midwives' participation in health policy development.

Theme	Subthemes	Sources
Societal factors	Power differential with physicians	(Perez et al. 2018; Hajizadeh et al. 2021; Radford and Maxwell 2022; Benton et al. 2020; Hajizadeh, Zamanzadeh, and Khodayari‐Zarnaq 2022)
	Poor public image of nursing and midwifery	(Hajizadeh et al. 2022, 2021; Radford and Maxwell 2022; Acheampong et al. 2021; Barzegar Safari, Bahadori, and Alimohammadzadeh 2020)
	Intersection between power, influence, and gender	(Hajizadeh et al. 2021; Radford and Maxwell 2022; Hajizadeh et al. 2021; Buse, Mays, and Walt 2012)
Work environment and organizational factors	Insufficient time	(Perez et al. 2018; Hajizadeh et al. 2021; Al Faouri et al. 2021; Anders 2021)
	Lack of organizational support for policy work	(Perez et al. 2018; Hajizadeh et al. 2021; 2022; Bar Yosef et al. 2020; Mattison et al. 2020)
	Insufficient access to education	(Perez et al. 2018; Hajizadeh et al. 2021)
	Limited access to resources	(Barzegar Safari, Bahadori, and Alimohammadzadeh 2020)
	Lack of communication	(Waddell et al. 2017)
Nursing and midwifery‐related factors	Not seen as a priority/lack of interest	(Hajizadeh et al. 2022, 2021; Barzegar Safari, Bahadori, and Alimohammadzadeh 2020)
	Lack of knowledge, skills, and awareness	(Perez et al. 2018; Hajizadeh et al. 2021; Anders 2021; AbuAlRub and Abdulnabi 2020; Bar Yosef et al. 2020; Barzegar Safari, Bahadori, and Alimohammadzadeh 2020; Longman et al. 2017; Shariff 2014)
	Challenging nature of policy work	(Perez et al. 2018; Hajizadeh et al. 2021)
	Limited exposure or experience	(Perez et al. 2018; Hajizadeh et al. 2021)
	Lack of strong mentors	(Perez et al. 2018; Hajizadeh et al. 2021)
	Lack of confidence	(Anders 2021)

One of the historically challenging barriers is related to societal factors. Most notably, this relates to the challenges of the professional position and the power of nursing and midwifery within health systems (Radford and Maxwell 2022). This theme is picked up by Ditlopo et al. (2014), who state nursing's and midwifery's proximity to and power differential with the medical profession as a major barrier to active participation in health policy.

Similarly, Shariff's (2014) research revealed a similar issue where the contribution of nurses in shaping health policy was largely overlooked, with doctors and other health professionals predominantly steering the course. The research highlights that nursing proximity to and power differential with physicians means that nursing influence and involvement are often overshadowed. Radford and Maxwell (2022) state that

relational identity to Medicine remains an Achilles heel for Nursing, which has yet to clearly articulate its unique and distinct contribution. (. 572)

The gendered nature of the nursing and midwifery professions has long been a barrier to full participation in health policy development. Although nursing and midwifery are predominantly female professions, gender dynamics continue to influence policymaking opportunities today. Numerous researchers have pointed out that women are often expected to conform to traditional gender roles, which limits their participation in leadership and policymaking (Bustelo 2017; Lombardo, Petra, and Verloo 2017). Gender biases have long been recognized as a factor restricting women's progression in leadership, particularly in health policy (Dhatt et al. 2017).

This was highlighted in the WHO report “Delivered by Women, Led by Men” (World Health Organization 2019), which found that women account for 90% of the nursing and midwifery professions but remain significantly underrepresented in leadership, management, and policy influence. Addressing these gendered power imbalances is crucial to ensuring that nurses and midwives, regardless of gender, have equal opportunities to shape health policy and advocate for their profession.

Nurses, as the largest segment of the healthcare workforce (World Health Organization 2020), play a crucial role in healthcare delivery and have unique insights into patient care and health systems. Yet, despite their critical role in shaping and implementing healthcare policies, their voices are often absent from global policy forums (Rasmussen et al. 2022).

The literature also highlights other forms of power imbalances, wherein nurses and midwives are perceived more as policy implementers rather than policymakers, particularly at the frontlines of healthcare (Etowa et al. 2023; Hajizadeh et al. 2021; Acheampong et al. 2021). This top‐down approach to health policy development marginalizes nurses and midwives, reinforcing hierarchical systems and cultural norms that limit their roles in policy formulation. Asuquo (2019) found that nursing and midwifery leadership in policy is often unrecognized, with their participation frequently restricted to data collection in research projects, rather than contributing to and engaging in research design and investigation. This marginalization and exclusion from health policy development make nurses and midwives feel overlooked and unacknowledged, leading to further disengagement and limited participation in policymaking (Hajizadeh et al. 2021; Acheampong et al. 2021).

These elements are imposing barriers to nursing and midwifery's participation in health policy development and may be interconnected to a range of other multifaceted causes. These can be broken down into two other main categories, including (a) the work environment and organizational factors and (b) nursing‐related factors (Hajizadeh, Zamanzadeh, and Khodayari‐Zarnaq 2022). It is important to acknowledge these categories are not mutually exclusive and are interconnected to produce a complicated web of issues.

The work environment and organizational factors is an overarching theme that refers to the physical and social conditions under which nurses and midwives perform their work. This includes the physical conditions, workload and role demands, interprofessional relationships, and the culture of an organization, governance, and the distribution of power.

Turale and Kunaviktikul (2019) believe that the work environment of nurses and midwives creates a lack of opportunities for participation in health policy development (Turale and Kunaviktikul 2019). This, in part, is caused by a range of factors, including being underrepresented in committees and decision‐making bodies. The causes of this are varied and potentially interconnected. For example, Hajizadeh et al. (2021) found that insufficient resources and the demanding nature of workloads leave nurses and midwives’ with limited time to engage in health policymaking (Hajizadeh et al. 2021). Al Faouri et al. (2021) also found that there was a lack of support and interest from policymakers in engaging with nurses (Al Faouri et al. 2021). Similar to this, Shariff (2014) found that lack of involvement was often associated with a top‐down approach to policy development, a negative image of the professions and the lack of available resources (Shariff 2014). There are several other reasons in the research; however, they contain similarities related to the above‐mentioned causes.

Separate from the work environment and organizational factors are nursing and midwifery‐related factors. These factors refer to the elements or issues that are intwined with the professions and affect professional practice. This includes elements such as education and continuing professional development, professional autonomy, and personal characteristics (Hajizadeh, Zamanzadeh, and Khodayari‐Zarnaq 2022).

One of the most common themes in the research associated with this area is related to the knowledge, skills, and attributes of nurses and midwives as it relates to health policy and policy development. In particular, the lack of education regarding health policy in undergraduate, postgraduate, and continuing professional development courses (Hajizadeh et al. 2021; Shariff 2014; Cohen et al. 2021; Al Faouri et al. 2021; Radford and Maxwell 2022; Kunaviktikul et al. 2019). The reasons for this included not being part of curricula (Radford and Maxwell 2022; Alluhidan et al. 2020) and lack of time and support to engage in continuing professional development (Lewinski and Simmons 2018; Hajizadeh et al. 2021). As Salvage and White (2020) point out, this can lead to nurses and midwives having “naïve assumptions” about health and healthcare as they cannot view issues through a sociopolitical lens. In addition, they point out that to actively influence and lead health policy, they need to understand more than the content related to a health issue, “but also the context, and the stakeholders and their interests” (. 3). These issues are closely related to the other factors identified, such as the feelings of being marginalized and powerlessness (Hajizadeh et al. 2021; Shariff 2014), concerns of speaking up due to fears of retribution and being ostracized, especially when their views challenge prevailing practices or the current authorities (Shariff 2014; Etowa et al. 2016; Donovan et al. 2012), and poor confidence due to a lack of experience and exposure in health policy development (Radford and Maxwell 2022; Hajizadeh, Zamanzadeh, and Khodayari‐Zarnaq 2022; Longman et al. 2017).

### 4.3 Nursing Research and Its Impact on Policy and Practice

Aboshaiqah and colleagues (2023) highlighted the critical role of nursing research in advancing nursing knowledge, improving patient outcomes, and informing policy and practice. However, the study found that nursing research in Saudi Arabia, like in many other countries, is still in its “early stages” of development. The literature review revealed that 63% of research participants were nurses, followed by 15.8% who were nursing students. Other healthcare providers made up 5% of the participants, while patients accounted for 7%. This indicates that much of the research is concentrated on the nursing workforce rather than on clinical practice and health outcomes.

The study underscores the need for a broader and more robust scope of nursing research, focusing on health outcomes and clinical practice, not just on the nursing workforce. Expanding research areas, including primary health care, will not only improve nursing practices but also significantly contribute to the development of health policies. Encouraging diversification of research will ensure comprehensive healthcare improvements and better patient outcomes.

### 4.4 The Commonly Identified Strategies to Improve Nursing and Midwifery's Influence on Health Policy

Research and discourse over the last ten years emphasize the critical need for strategies to enhance the involvement and influence of nursing and midwifery in health policy development. Key strategies include enhancing education, continuing professional development, leadership and mentorship programs, access to resources, and active participation in professional organizations (see Table 3).

**TABLE 3:** Strategies identified in the literature that increase nursing and midwifery's involvement in health policy development.

Source	Country	Research	Strategy	Findings/outcomes	Recommendations
(Hajizadeh et al. 2022)	Iran	Mixed method	Comprehensive framework to address political, cultural, economic, and social factors	Education alone does not increase participation in health policy development	Each country should develop a framework to address the political, cultural, economic, and social factors that impact health policy development
(Lee and Choi 2022)	Multiple	Literature review	Review of education strategies to encourage participation in health policy for nurses	Health policy education increases the competency and interest of nurses and nursing students in policy participation	Health policy should be made a compulsory part of nursing education. Faculty staff require knowledge and experience in health policy
(Rosa et al. 2022)	Multiple	Literature review	Review of strategies to advance universal palliative care	Nurses should be involved in associations to increase their participation in health policy development	Increase engagement and participation in professional associations, interdisciplinary, intersectoral, and community partners
(Rumsey et al. 2022)	Pacific Island Countries	Qualitative	A forum for government chief nurses and midwives	Improved influence on local and regional health policies by senior leaders	Countries across specific regions should consider adopting similar networks of government senior nurses and midwives
(Ferrada‐Videla et al. 2021)	Canada	Qualitative	Support for building strategic leadership	There is a need to build strategic leadership capabilities through continuing professional development	Improve continuing professional development to include a focus on political skills, emotional intelligence, and managerial skills
(Hajizadeh et al. 2021)	Multiple	Systematic review	Systematic review of the literature to identify strategies across the Eastern Mediterranean, African, and Americas regions	Nurses’ participation in health policy development requires strategies to address management and organizational factors	Education across multiple domains and nursing research is required to increase nurses’ participation in health policy development
(Hudson et al. 2021)	Multiple	Literature review	Review of the effectiveness of online learning strategies to improve leadership and policy participation for nursing students	Unable to assess the effectiveness of the online learning programs on nursing students’ involvement in health policy	Further research is required on the impact and effectiveness of online resources related to leadership and health policy
(Mattison et al. 2021)	Multiple	Literature review	Framework to strengthen midwifery associations	Midwifery associations are key enablers to strengthening their profession across political, health, and education systems	Invest in midwifery associations to increase influence in health policy
(Wolbers et al. 2021)	Netherlands	Mixed method	Leadership education program and mentorship	Self‐perceived increased participation in health policy development	Offer continuing professional development in leadership
(Han 2020)	Korea	Qualitative	Promotion of social responsibility to participate in health policy development and improvement of solidarity amongst nurses	Improved engagement in health policy design and development	A mixed approach that improves awareness of social responsibility, knowledge acquisition, understanding of healthcare policy problems, social solidarity, and improved competence
(Gandelman and Moran 2021)	Israel	Qualitative	Improving access to professional resources	Increased awareness and interest in the development of health policy	Include health policy education in nursing curricula and continuing professional development
(Wichaikhum et al. 2020)	Thailand	Qualitative	Implementation of multiple strategies to build competence, increase opportunities and improve awareness	For nurses to have increased involvement in health policy development requires skills in leadership, networking, and health policy. Other barriers to nurses participating in health policy must be addressed.	Multiple strategies are required to improve participation in health policy development
(Horton et al. 2019)	USA	Qualitative	Simulated learning	Increased confidence to participate in health policy development	Incorporate public health policy in nursing curriculum
(de Cordova et al. 2019)	USA	Qualitative	Incorporation of advocacy and health policy competencies in university curricula	Improved consistency between education programs in the quality of health policy courses benefits students’ confidence in participating in health policy	Integrate health policy competencies into undergraduate and postgraduate nursing programs
(Lavenberg et al. 2019)	USA	Mixed methods	Review of the impact of an evidence‐based practice center on nursing policy	An evidence‐based practice center increases nursing involvement and influence in health policy development	Health systems should establish an evidence‐based practice center to increase and improve the quality of nursing input into health policies
(Salvage et al. 2019a)	Multiple	Editorial	Global Nursing Leadership Institute program supporting “top” nurses from around the world	Improved networking between senior nurses around the world. Improved confidence in participating in health policy	Increase investment in strategic nursing leadership programs.
(Thompson and Schwartz Barcott 2019)	USA	Systematic review	Review of the benefits of nurse scientists as a knowledge broker	Nurse scientists improve the translation of science into health policy	The nurse scientist role should be promoted worldwide to support nursing's influence in health policy
(Lewinski and Simmons 2018)	USA	Qualitative	Continuing Professional Development short course for nurses	Increased interest in participating in health policy development	Increase the amount of resources for nurses to participate in health policy development
(Perez et al. 2018)	USA	Qualitative	Multiple strategies, including continuing professional development programs, mentorship, and addressing organizational support	Comprehensive approach required to improve nursing's involvement in health policymaking	Multiple strategies required, including nursing competence, improving alignment between nursing research and health policy, and fostering development in communication
(Perry and Emory 2017)	USA	Qualitative	Educational presentations to undergraduate and postgraduate students	Increased confidence to participate in health policy development	Incorporation of practical learning into all levels of nursing education programs
(Waddell et al. 2017)	USA	Qualitative	Education in communication skills and sociopolitical knowledge	Increased confidence in participation in health policy development	Advance nurses' understanding of health policy at all levels of nursing education
(Woodward et al. 2016)	USA	Literature review	Understanding of modifiable factors that support nurses to engage in politics and health policy	Modifiable factors identified to increase nurses’ involvement in health policy require knowledge, interest, and networking	Increased participation requires multiple strategies, including (a) integration of politics and policy in nursing education, and (b) provision of information to increase personal interest, (c) membership in professional and advocacy organizations
(Lopes et al. 2015)	Multiple	Qualitative	Review of the involvement of midwifery associations in policy development	Midwifery associations are ideally placed to support policy development as a result of data, evidence, and knowledge	Midwives can increase their policy involvement by joining professional associations

### 4.5 Motivations for Participation

One of the most important and simplest tenets addressed is the motivations for nurses and midwives to participate in the health policymaking process. Hajizadeh et al. (2021) identify several motivations for nurses to engage in health policy. These include a professional commitment to improving healthcare quality and safety, close proximity to patients and families across various settings, direct impact of policies on nurses, and the potential to contribute to effective health policies.

### 4.6 Enhancing Knowledge, Skills, and Experience

Many studies highlight insufficient knowledge, skills, and experience in health policy as primary barriers to nursing and midwifery involvement. The overwhelming number of strategies focuses on improving access to education on leadership and health policy. This includes incorporating health policy into undergraduate and postgraduate education programs (Lee and Choi 2022; Gandelman and Moran 2021; Horton et al. 2019). Additionally, providing resources and time for continuing professional development (Ferrada‐Videla et al. 2021; Wolbers et al. 2021) and ensuring that educators have the necessary skills and experience to teach health policy (Hudson et al. 2021; Lee and Choi 2022) are essential. Standardizing competencies in undergraduate curricula (de Cordova et al. 2019) and supporting learning through mentorship programs (Wolbers et al. 2021) also play a significant role. These strategies appear to have improved the confidence and willingness of nurses and midwives to participate in health policymaking.

### 4.7 Confidence and Willingness to Participate

Lewinski and Simmons (2018) and Hajizadeh and colleagues (2021) found that although education increased nurses’ and midwives’ confidence and willingness to engage in health policy development, it did not necessarily lead to greater involvement in policy and advocacy activities. The researchers view education as a crucial step toward empowerment and participation in health policy. However, they believe that inadequate participation will persist as long as societal, management, and organizational barriers remain unaddressed. They recommend tackling these determinants and developing a comprehensive framework that incorporates multiple strategies to enhance nursing and midwifery's involvement in health policy and advocacy.

### 4.8 Role of Professional Organizations

Membership in professional organizations is widely recognized as a key strategy for enhancing the influence of nursing and midwifery in health policy development. Research suggests that collective action within such organizations enables nurses and midwives to increase their visibility and advocacy power, thereby attracting greater attention from policymakers compared with less coordinated groups (Woodward et al. 2016). Effective collaboration within these organizations, however, requires a focus on specific issues, objective problem‐solving, and the ability to navigate complex, diverse perspectives diplomatically. When these conditions are met, significant policy changes can be achieved, as evidenced by Han (2020), who highlighted the role of nurses joining “civic groups” to effectively advocate for health policy reform. Such involvement allows nurses to gain valuable political experience, broaden their influence, and mobilize action for systemic change (Han 2020).

### 4.9 Effective Strategies for Health Policy Development

Chiu et al. (2023) interviewed executive leaders of professional nursing associations, emphasizing the critical role of nursing in health policy, particularly during the COVID‐19 pandemic. During the pandemic, Professional Nursing Associations were key advocates for better public health measures, accurate information dissemination, and support for vulnerable populations. Chief nurses and professional nursing bodies advised governments on improving pandemic responses and broader health system reforms, positioning themselves as essential contributors to health policy.

To increase nurses’ input into health policy development, Chiu and colleagues (2023) identified several effective strategies. This included maintaining connections with decision‐makers, translating evidence and nursing experiences into policy solutions, and advocating for the establishment of formal leadership roles for nurses. The recommendations focus on fostering collaboration among professional nursing associations, governments, and other stakeholders to ensure comprehensive and well‐informed policy responses. Additionally, they highlighted the importance of promoting a strong nursing research culture, enhancing publication output, and securing funding to support nurse researchers.

### 4.10 Challenges in Collaboration

The collaboration and uniting of health professionals, however, do not always result in involvement of local, subnational, and national health policy development. For example, Lopes et al (2015) found that less than two thirds of associations reported involvement in policy and planning. In addition, they found that many professional associations were often unaware of their country's approach to human resources for health and that their roles in policy development were “quite limited.” The causes identified by the authors include lack of competency in advocacy, resource mobilization, and leadership and management skills within the membership associations.

## 5 Discussion

Three key areas became apparent after careful review of the literature. These are:

- The insufficiency of the current approach to increasing nursing and midwifery's participation in health policy
- The narrow focus and assumptions of health policy research
- The need for empirical assessment of nursing and midwifery's role in health policy formulation.

The literature consistently indicates that nurses and midwives are underrepresented in health policymaking, which is a long‐standing issue that has not improved significantly over time. Benton et al (2020) state that there has been an increase in the number of calls for nurses and midwives to become increasingly involved in policy development and politics. Much of this focus has been on the education of nurses and midwives, improving their understanding of policy and political processes. However, this focus on education has not necessarily translated into increased involvement of nurses and midwives in health policy development. This is evidenced in the plethora of articles, white papers, and reports stating that nurses and midwives are not adequately represented at the policy table. These findings suggest that challenges persist in effectively integrating nurses and midwives into policy decision‐making.

While advocating for education at all levels for nurses and midwives is crucial, it alone is unlikely to significantly increase participation in health policy, except for a few exceptional or fortunate individuals (Radford and Maxwell 2022). The literature emphasizes that addressing this issue requires being part of a collective and united group. Membership in professional organizations can enhance nursing and midwifery representation in the broader health policy arena, but it does not guarantee that their voices will be heard. This was evident in the work of Lopes and colleagues (2015), who identified numerous professional organizations that were not participating in local or national policy and planning due to either a lack of awareness of ongoing work or exclusion by health policymakers.

Shiffman et al. (2016) examined this lack of engagement in health policy by investigating the emergence and effectiveness of global health networks, analyzing why some organizations excel while others lag behind. They identified several key factors influencing success, including governance mechanisms, strong leadership, and effective coordination. Successful organizations typically had a clear understanding of the problem, effectively mobilized support, and presented united solutions. In contrast, organizations that struggled to achieve their desired outcomes often had competing priorities, leading to confused responses to health policies and vested interests that did not represent their membership. Building on Shiffman's work, White (2018) specifically addressed the nursing field, citing insularity, lack of coalition building, scarce resources, and political naivety as significant factors limiting the effectiveness of nursing organizations in influencing health policy.

The ongoing limited involvement of nurses and midwives is a critical concern. Despite the consistent calls for increased nursing and midwifery involvement in policy development and politics, the current strategies, especially those centered on education and collective action, have proven insufficient in bridging this gap. Moving forward, it is imperative to explore and implement new strategies that address these highlighted factors. By developing effective strategies, nursing and midwifery can reposition to be a forceful and effective voice in health policy development, advocating not just for the profession but, more importantly, for the broader health and wellbeing of the communities they serve.

In the landscape of research over the last ten years on nursing influence on health policy development, there is significant concern regarding its quality and gaps in understanding. A noticeable trend is the emphasis on the experiences of individual actors, primarily nurses and midwives (Chiu et al. 2023; Aboshaiqah et al. 2023), which does not furnish a comprehensive understanding of the health policy landscape. A focus such as this implicitly assumes that health policies are devised through logical and meaningful processes, which contradicts the often political and complex reality of policy formulation, as highlighted by O'Brien et al. (2020).

Walt and Gilson's (1994) work on developing effective health policy underscores the need to understand and consider four critical elements: content, processes, context, and actors. The literature, however, tends to focus on the “actor” component with a disproportionate emphasis on nurses and nursing organizations. As a result of this singular perspective, crucial aspects like the content of the policy, the processes through which is developed and implemented, and the context in which it exists are left out. This means that the nuances of policy development might be left out such as the sociopolitical and economic conditions. Therefore, whilst there might be some gain in understanding the challenges and issues related to the actors (i.e., nurses and midwives), there is a lack of comprehensive understanding of the full range of factors influencing effective health policy development and implementation.

Similarly, the research primarily focuses on a single group of human actors (i.e., nurses and midwives) whilst leaving out the vast array of others such as policy makers, other health professionals, patients, and the community. In addition to human actors, the non‐human actors (e.g. regulations, guidelines, institutions, and technology) are also left out of the research. Such omissions result in a narrow and potentially skewed picture of the policymaking environment.

The actor network theory (Latour 2007) provides an appropriate methodology to redress this imbalance. The actor network theory focuses on the interconnected network of human and non‐human actors. This can be used to explore the relationships between actors, and this influences health policy development. This contrasts with the current approach of viewing actors in isolation. As such, actor network theory could provide further illumination into the barriers nurses and midwives face, their specific needs, and their unique contributions. Health policymaking is a complex process with multiple interplaying factors and actors. It requires balancing diverse interests, managing resources, understanding societal norms, and navigating political landscapes.

Through the adoption of actor network theory (Latour 2007), researchers could explore how various actors (both human and non‐human) are interconnected with each other and how they mutually influence one another. For example, one could investigate how nurses and midwives interact with policymakers or examine how a specific health technology impacts the roles and relationships among actors. Actor network theory brings attention to the fact that actors do not operate independently but rather exist within a network of relationships that both shape and are shaped by the policymaking process. Research undertaken in this way will likely expand on the current knowledge and provide valuable insights into the complexities associated with the barriers and enablers with health policymaking.

Additional factors contributing to the research gaps pertain to the ability to quantitatively assess nursing and midwifery's involvement in health policy formulation. Existing literature frequently delves into the theme of limited nurse and midwives’ participation in policymaking but fails to provide an empirical analysis of actual participation rates. Consequently, it remains uncertain whether health policies are crafted entirely devoid of nursing and midwifery contributions or if there is merely a partial lack of their involvement. This ambiguity leads to potential inaccuracies in assertions about nursing's and midwifery's engagement in policy development.

In parallel to this issue is the scarcity of studies showcasing tangible enhancements in nursing and midwifery's engagement in policy formulation, after the design and implementation of specific improvement strategies. While many published articles do indicate certain benefits arising from the application of strategies such as education, the outcomes are primarily linked to increased awareness, confidence, and knowledge among nurses and midwives. However, these studies often do not extend to evidence of augmented participation and involvement in policymaking. This lack of demonstrable results limits the confidence in the effectiveness of strategies designed to address the challenges and issues associated with policy development.

Future research should strive for a more empirical approach, quantitatively measuring nursing and midwifery's participation in health policy development, while also addressing the qualitative aspects of such involvement. Moreover, there is a pressing need for research that evaluates the effectiveness of strategies intended to enhance nursing and midwifery's participation, not only in terms of awareness and knowledge but also in terms of actual engagement in policymaking processes. By filling these research gaps, the field can gain a more comprehensive understanding of nursing's and midwifery's role in health policy development and improve strategies to enhance this critical involvement.

## 6 Implications for Nursing and Health Policy

The findings from this review highlight the need for a broader, more nuanced approach to research on nursing and midwifery's involvement in health policy. Current literature often focuses on the individual experiences of nurses and midwives, while overlooking the broader policy development context, including other key actors and systemic factors. A comprehensive understanding of health policy formulation must incorporate the roles of policymakers, other health professionals, and non‐human actors such as regulations and technologies. Future research should embrace this complexity and adopt frameworks, such as actor network theory, to explore how various actors interact within the policy process. By considering the full range of factors influencing policy, a more effective understanding of the barriers and enablers of nursing's involvement can be achieved, leading to improved strategies for participation.

Addressing the underrepresentation of nurses and midwives in policy development requires more than just education, though this remains a crucial starting point. Strategies must include the integration of health policy into nursing curricula at all levels, provision of ongoing professional development, and strengthening involvement in nursing associations. Professional organizations must also be empowered to serve as platforms for collective action, increasing their advocacy power and enhancing their ability to influence policy. However, education and organizational involvement alone are insufficient. A comprehensive, multifaceted approach is needed to tackle the barriers identified in the literature, including power imbalances, gender dynamics, and lack of institutional support.

Ultimately, the shift from nursing being perceived as invisible in health policy to being recognized as an invaluable contributor requires a concerted effort to address these systemic barriers. By developing and implementing new strategies that address these challenges, nursing and midwifery can assert their essential role in shaping health policy, ensuring that policies are not only informed, but also driven, by the insights of frontline healthcare providers.

## 7 Limitations

The exclusion of non‐English articles may result in a language and geographic bias, limiting the global scope of the findings. Future research would benefit from multilingual perspectives to provide a more comprehensive understanding of nursing and midwifery roles in health policy across the world.

## 8 Conclusions

This literature review has underscored the critical role that nurses and midwives can play in shaping health policy and influencing health systems worldwide. This influence, however, is often undermined due to societal, individual, management and organizational factors that limit their capacity to contribute meaningfully to policy development. Transformative changes are needed to empower nurses and midwives to be more actively involved in policy development.

The literature emphasizes the importance of improving access to education and continuing professional development to enhance nurses’ and midwives’ engagement in health policy. These strategies have been shown to boost awareness, confidence, and knowledge. Additionally, involvement in professional organizations is recommended to increase their policy influence. However, the findings also indicate that these strategies alone are insufficient to fully address the issue of nursing and midwifery's involvement in health policy.

Despite these findings, significant gaps remain in the literature, particularly regarding the broader array of actors involved in policymaking and the sociopolitical and economic contexts in which policies are developed. Existing research often focuses on individual experiences, predominantly those of nurses or midwives, neglecting these broader factors. Addressing these gaps through future research is crucial.

Future research should aim to develop strategies and frameworks that harness the unique insights and innovative potential of nurses and midwives. By doing so, policymakers can better leverage their contributions to drive health system transformation, address pressing health challenges, and deliver high‐quality care for all.

## Author Contributions

Study design: DS, KB, JB, and JW. Data collection: DS. Data analysis: DS, KB, JB, and JW. Study supervision: KB, JB, and JW. Manuscript writing: DS. Critical revisions for important intellectual content: KB, JB, and JW.

## Ethics Statement

This study did not involve primary data collection or interactions with participants, as it is based on a literature review of publicly available research. Therefore, no ethics approval was required. No participants were involved, and no consent—written or verbal—was necessary for this study.

## Conflicts of Interest

The authors declare no conflicts of interest.

## Declaration of Artificial Intelligence

During the preparation of this work, the authors used ChatGPT in order to improve the readability of the manuscript. After using this tool/service, the authors reviewed and edited the content as needed and take full responsibility for the content of the published article.

## Endnote

1 Original research had a greater number of articles that were reviewed and assessed. Due to the limitations of the number of references, these were removed from the article and are available on request. The omitted articles largely duplicated the findings and were >5 years at the time of review.
